# Estimated Effects of Asian Dust Storms on Spatiotemporal Distributions of Clinic Visits for Respiratory Diseases in Taipei Children (Taiwan)

**DOI:** 10.1289/ehp.1104417

**Published:** 2012-03-15

**Authors:** Lung-Chang Chien, Chiang-Hsing Yang, Hwa-Lung Yu

**Affiliations:** 1 Department of Internal Medicine, Division of Health Behavior Research, Washington University School of Medicine, St. Louis, Missouri, USA; 2 Department of Health Care Management, National Taipei University of Nursing and Health Sciences, Taipei, Taiwan; 3 Department of Bioenvironmental Systems Engineering, National Taiwan University, Taipei, Taiwan

**Keywords:** Asian dust storm, children’s clinic visit, respiratory disease, spatiotemporal analysis

## Abstract

Background: Increases in certain cause-specific hospital admissions have been reported during Asian dust storms (ADS), which primarily originate from north and northwest China during winter and spring. However, few studies have investigated the relationship between the ADS and clinic visits for respiratory diseases in children.

Objective: We investigated the general impact to children’s health across space and time by analyzing daily clinic visits for respiratory diseases among preschool and schoolchildren registered in 12 districts of Taipei City during 1997–2007 from the National Health Insurance dataset.

Methods: We applied a structural additive regression model to estimate the association between ADS episodes and children’s clinic visits for respiratory diseases, controlling for space and time variations.

Results: Compared with weeks before ADS events, the rate of clinic visits during weeks after ADS events increased 2.54% (95% credible interval = 2.43, 2.66) for preschool children (≤ 6 years of age) and 5.03% (95% credible interval = 4.87, 5.20) for schoolchildren (7–14 years of age). Spatial heterogeneity in relative rates of clinic visits was also identified. Compared with the mean level of Taipei City, higher relative rates appeared in districts with or near large hospitals and medical centers.

Conclusion: To our knowledge, this is the first population-based study to assess the impact of ADS on children’s respiratory health. Our analysis suggests that children’s respiratory health was affected by ADS events across all of Taipei, especially among schoolchildren.

Evidence of adverse health effects resulting from ambient air pollutants, including increased hospitalization and mortality rates, has been accumulating in recent decades (Dockery et al. 1993; Pope et al. 2002). In Taiwan, high ambient air pollution levels are often closely associated with the incidence of Asian dust storms (ADS), which occur about four to five times a year (primarily in the winter and spring). ADS originate from arid areas of northwest and north China and Mongolia and spread to countries in East Asia (Liu et al. 2006). It has been reported that concentrations of crustal elements and sea salt species in PM_2.5–10_ (particulate matter ≤ 2.5 to 10 µm in diameter) during ADS episodes in central Taiwan may be twice as high as concentrations during non-ADS periods (Cheng et al. 2005). Higher concentrations of PM were also measured during ADS in Korea and Japan, and higher concentrations of other elements such as aluminum, iron, and calcium were measured in PM during ADS events in China (Choi et al. 2001; Ma et al. 2001; Zhou et al. 1996).

Concerns about health impacts due to changes in concentrations, size distributions, and chemical compositions of ambient pollutants during dust storms have been raised in recent years. Studies have reported significant increases in daily mortality and hospital admissions from all causes within 2 days after dust episodes (Chen et al. 2004; Middleton et al. 2008) as well as significant increases in emergency visits for pneumonia, ischemic heart diseases, and cerebrovascular diseases during dust events (Chan et al. 2008). However, changes in hospital admissions for cardiopulmonary diseases during and after the dust storms are less clear (Bell et al. 2008). Positive associations have been reported between dust storm events in Taipei and hospital admissions or clinic visits for asthma, stroke, congestive heart failure, and conjunctivitis (Yang 2006; Yang et al. 2005a, 2005b, 2009). Most studies of the health impacts of dust storms in Taiwan have been based on hospital admission data. Therefore, inferences may be limited to vulnerable populations with serious defects in cardiopulmonary function (Bell et al. 2008; Middleton et al. 2008; Yang 2006; Yang et al. 2005a, 2009). Children have been considered to be particularly sensitive to ambient pollutant exposures (Bates 1995; Berhane et al. 2004; Salvi 2007; Schwartz 2004), but relatively few studies have focused on the health impacts of dust storms in children. However, it has been reported that reductions in peak expiratory flow rate in schoolchildren was more strongly associated with ambient heavy metals than with PM_2.5_ levels after dust storms (Hong et al. 2010).

To understand the general impact of ADS on children’s respiratory health, we used population-based data on daily clinic visits for respiratory diseases (all causes combined) registered in hospitals and clinics in Taipei City during 1997–2007. In addition to investigating temporal patterns in relation to ADS, we also investigated the spatial distribution of clinic visits across 12 districts in Taipei City using the structured additive regression (STAR) model (Brezger et al. 2005). The STAR model is based on a Bayesian framework that can accommodate both linear and nonlinear explanatory variables as well as spatial and temporal autocorrelations.

## Materials and Methods

*Health care utilization data.* Taiwan began its National Health Insurance (NHI) program in March 1995. By the end of 1996, the Bureau of National Health Insurance (BNHI) had contracted with > 97% of hospitals and clinics nationwide (BNHI 2000), and 96% of Taiwanese residents were enrolled in the program (Lu and Hsiao 2003). The National Health Research Institutes (NHRI) has cooperated with the BNHI to establish a research database using standard procedures to assure the quality and accuracy of claim data (Tseng 2004). For the purposes of personal privacy and confidentiality, all individually identifiable health information of the database is encrypted by the BNHI before its release. The database includes ambulatory care expenditures by visits and appointments for contracted medical facilities. Procedure and diagnostic codes are used to retrieve cause-specific data according to diagnosis-related groups or the *International Classification of Diseases, 9th Revision, Clinical Modification* (ICD-9-CM) (Centers for Disease Control and Prevention 2006) classification codes. For this study we obtained a database that included the hospital location and appointment date for all clinic visits for respiratory diseases [ICD-9-CM codes 460 to 519, or A-code A31 to A32 (A-codes from the abridged version)] in children ≤ 14 years of age in Taipei City during 1997–2007. These clinic visits data were additionally categorized according to ages of preschool children (0–6 years of age) and schoolchildren (7–14 years of age) for more detailed analyses.

**Figure 1:**
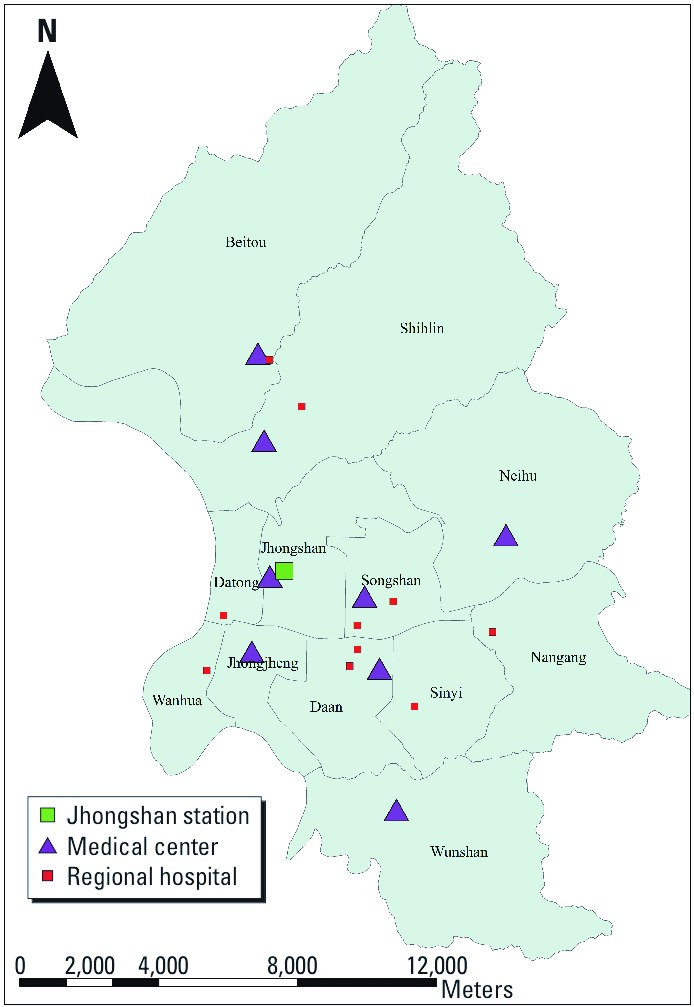
Geographic distribution of the districts, the medical centers, and the Jhongshan air quality monitoring station in Taipei City.

*Environmental data.* ADS events during 1997–2007 were identified by the Department of Atmospheric Science at Chinese Culture University (CCU) from 1997 to 2000 and the Taiwan Environmental Protection Agency (TWEPA) from 2001 to 2007 (Table 1). Specifically, an identified ADS event in the CCU database should satisfy both criteria: *a*) the visibility at any three neighboring First GARP (Global Atmospheric Research Programme) Global Experiment–type ground stations (Nee et al. 2007) in East Asia is < 1 km for 24 hr; and *b*) at least one of the air quality monitoring stations (Wanli, Guanyin, Danshui, and Yilan stations) observed PM_10_ concentrations > 100 µg/m^3^ (Yu and Liu 2003). After 2000, the TWEPA used three steps to define an ADS event officially. First, TWEPA confirmed the occurrence of ADS in China or Mongolia by checking a Weather Integration and Nowcasting System developed by Taiwan Central Weather Bureau (Chen 2002). Second, TWEPA estimated the ADS transport by using Moderate Resolution Imaging Spectroradiometer remote sensing data (National Aeronautics and Space Administration 2012) and several ADS models to forecast whether the ADS will come to Taiwan. If forecasting results show that an ADS may come to Taiwan, the TWEPA would finally announce an ADS day as long as at least one of the four air quality stations (the Matsu, Wanli, Guanyin, and Yilan stations) measured PM_10_ concentrations > 100 µg/m^3^ (TWEPA 2011). In addition to the ADS data, PM_10_ concentrations have been regularly monitored at TWEPA stations across Taiwan since 1994. Both PM_10_ concentrations and temperature measurements used in our analysis were based on daily observations at the Jhongshan monitoring station located in the most populated area of Taipei City (Figure 1).

**Table 1:** Dust storm days in Taipei, 1997–2007.

Year	Date (month/day)
1997		1/1, 3/7–3/8, 3/30, 4/8, 4/21, 4/27–4/28,
1998		1/4, 2/13, 2/18–2/19, 3/7, 3/19, 3/30, 4/4, 4/15, 4/17–4/19, 4/24–4/26, 5/1, 11/5, 12/15
1999		1/27, 2/19, 3/8–3/9, 3/26, 4/7, 4/13, 11/25
2000		3/6–3/7, 3/24–3/25, 3/28–3/29, 4/6, 4/8, 4/10–4/11, 4/15–4/16, 4/22, 4/27–4/28, 5/1, 5/3–5/4, 5/13–5/18, 12/24
2001		1/13–1/15, 2/1, 2/16–2/17, 2/21–2/25, 3/1–3/7, 4/12–4/14, 5/1–5/2
2002		2/11–2/12, 3/6–3/9, 3/23–3/24, 3/31–4/1, 4/8–4/15, 4/17–4/19
2003		2/18–2/19, 2/23–2/25, 3/6–3/9, 3/25–3/30, 4/25–4/28
2004		1/1–1/4, 1/13–1/14, 1/21–1/22, 1/24–1/25, 2/6–2/12, 2/14–2/16, 2/26–2/27, 3/3–3/7, 4/2–4/4
2005		3/18–3/19, 11/29–11/30, 12/21–12/22
2006		3/19–3/20, 3/29–3/30, 4/20–4/21
2007		1/28–1/29, 4/2–4/3, 4/17–4/18, 12/30–12/31
The ADS days are obtained from the CCU and TWEPA data sets for 1997–2000 and 2001–2007, respectively. The details of the ADS definition appear in “Environmental data.”		

*Study Area.* Taipei City, located in Northern Taiwan, is the capital and largest metropolitan area in Taiwan, with a population of > 2 million. Taipei is bounded by the Yangming Mountains to the north, the Linkou mesa to the west, and the Snow Mountains to the southeast, resulting in a basin topography that reduces air diffusion and increases concentrations of ambient air pollutants. We analyzed clinical visit data from 12 districts in Taipei City. The geographic distribution of major medical centers and hospitals is also shown in Figure 1.

## Methods

**Figure 2:**
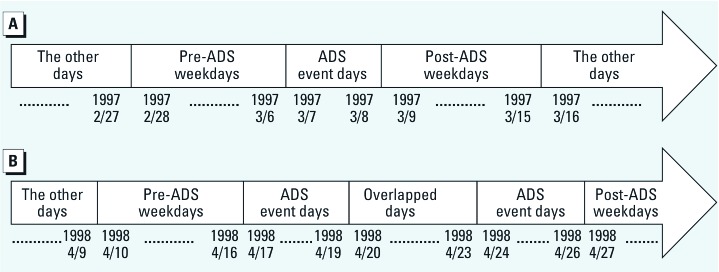
Schematic diagrams of the definitions for pre-ADS weekdays, ADS event days, post-ADS weekdays, and the other days (month/day). (*A*) Pre- and post-ADS weekdays are not overlapped; (*B*) pre- and post-ADS weekdays are overlapped.

We used the STAR model to account for temporal autoregressive correlation and spatial autocorrelation in daily clinic visits for respiratory diseases in children ≤ 14 years of age among the 12 districts in Taipei City during 1997–2007. The outcome *Y_st_* is the number of children’s clinic visits for respiratory diseases each day in district *s* (*s* = 1, 2, ..., 12) on day *t* (*t* = 1, 2, ..., 4,017). We assumed that daily clinic visits followed a Poisson distribution with expectation E(*Y_st_*) = µ*_st_* and variance Var(*Y_st_*) = ϕ*_s_*µ*_st_*, where ϕ*_s_* is an overdispersion parameter indicating the variation in clinic visits that is not explained by the predictors. We used six dummy variables for day of the week (DOW) from Monday through Saturday (i.e., Sunday is the reference level) to adjust for short-term trends, and a continuous variable of PM_10_ at time *t* (*PM_t_*) to adjust for the confounding effect of air pollutant. We also modeled two nonlinear variables, *T* for calendar time and *TP_t_* for daily mean temperature at time *t*, using smoothers to adjust for long-term trends and the effects of weather. To estimate the influence of ADS events on children’s respiratory clinic visits, we classified all days during the study period as ADS event days, pre-ADS weekdays (i.e., the 7 days before the first day of an ADS event), post-ADS weekdays (the 7 days after the last day of an ADS event), or the other days. For example, in the second ADS event of 1997 from March 7 to March 8 (3/7 to 3/8), the pre-ADS weekdays were considered to be 1 week before 3/7, and the post-ADS weekdays were considered to be 1 week after 3/8, as shown in Figure 2A. In case two consecutive ADS events were too close (e.g., in 1998, the 9th ADS from 4/17 to 4/19 and the 10th ADS from 4/24 to 4/26), and caused the post-ADS weekdays of the previous ADS to overlap the pre-ADS weekdays of the next ADS, as illustrated in Figure 2B, the overlapped days were also defined as the ADS event days.

Next, a vector DSI containing three dummy variables of dust storm index for ADS event days, post-ADS weekdays, and the other days (i.e., pre-ADS weekdays are the reference level) was created to analyze the ADS influence. Therefore, the complete STAR model can be described by the equation

log(µ*_st_*) = α + δ × (DOW) + β × DSI + γ(*PM_t_*) + f(*TP_t_*) + f(*T*) + f_spat_(*s*) + offset*_s_*, [1]

where α represents the overall log relative rate for all districts, δ is a 1×6 vector containing the coefficients of the day of the week variables, β is a 1×3 vector containing the coefficients of the dust storm index variables, and γ is the coefficient of PM_10_. The temperature smoother f(*TP_t_*) and time smoother f(*T*) are modeled using natural cubic B-splines with a second-order random walk penalty (Lang and Brezger 2004). The offset is the logarithm of the district-level population based on the 2000 Census (Taiwan Ministry of Interior 2000).

The spatial function f_spat_(*s*) in Equation 1 can be decomposed into an unstructured spatial effect and a structured spatial effect. The unstructured spatial effect can be regarded as a random intercept fitted by an exchangeable normal prior with mean zero and variance σ^2^_u_. The structured spatial effect is a Markov random field (Kindermann and Snell 1980), which is achieved by assuming a normally distributed conditional autoregressive prior with mean Σ*_s_*_´∈Θ_*_s_* η*_s_*_´_*/N_s_* and variance σ^2^*_s_*/*N_s_*. The denominator *N_s_* in both mean and variance is the number of neighboring districts adjacent to district *s*, and *s*´∈Θ*_s_* indicates that district *s*´ belongs to one of the neighboring districts Θ*_s_* of district *s*. The nominator η*_s_*_´_ in mean represents the structured spatial effect in district *s*´ (Fahrmeir and Lang 2001). Variances and unknown smoothing parameters are estimated simultaneously with hyper-priors assigned by inverse gamma distributions [IG(0.001, 0.001)]. The structured spatial effect allowed us to estimate the relative rate (RR) in district *s* compared with the mean value for the population as a whole, accounting for spatial autocorrelation (Fromont et al. 2010). Moreover, maps of structured spatial effects at the district level were established to visualize the geographic distribution of RR. Spatial effects were also classified into three groups according to their posterior probabilities with respect to the number 1: *a*) 80% of the posterior distribution < 1 indicating a significantly lower RR than the mean level for the Taipei City area; *b*) 80% of the posterior distribution > 1 corresponding to a significantly higher RR than the mean level for the Taipei City area; *c*) the other districts corresponding to a nonsignificant difference of RR compared with the mean level for the Taipei City area (Adebayo and Fahrmeir 2005; Kandala et al. 2011; Kazembe 2009; Kazembe and Mpeketula 2010; Kazembe et al. 2008).

The STAR model is fitted by a fully Bayesian influence approach using a Markov Chain Monte Carlo technique carried out by randomly drawing samples from a fully conditional distribution of blocks of parameters given the rest of parameters and data (Musio et al. 2010). In the random sampling scheme, 22,000 iterations are carried out, with the first 2,000 samples discarded. Every 20th sample is stored from the remaining 20,000 samples, giving a final sample of 1,000 for posterior estimations. Also, corresponding 95% credible interval (CI) was determined based on the posterior distribution of the 1,000 samples, and an estimate was considered significant if its 95% CI did not include zero. The data analysis was implemented by the BayesX 2.01 software package (Belitz et al. 2009).

## Results

The major categories of children’s respiratory diseases in our data set were upper respiratory infection (70%), lower respiratory infection (5.3%), asthma (4.7%), and chronic obstructive pulmonary disease (0.2%). Daily average respiratory clinic visits for children varied among 12 districts in Taipei City before, during, and after ADS events from 1997 through 2007 (Table 2). Daily mean (± SD) visits during ADS event days ranged from 419.9 ± 156.2 in the Datong District to 1550.5 ± 492.4 in the Shihlin District. Most districts had lower daily averages during pre-ADS weekdays, except for the Datong, Beitou, and Wunshan Districts. Daily average visits during post-ADS weekdays were higher than during ADS days in all districts except for the Jhongjheng District, in which the average decreased from 945.4 ± 348.1 to 930.1 ± 359.6. The greatest increase in visits from pre-ADS weekdays to ADS event days occurred in the Jhongjheng District (27.3 visits), and the greatest increase in visits from ADS event days to post-ADS weekdays was observed in the Wunshan District (37.3 visits).

**Table 2:** Daily average clinic visits for respiratory disease among children ≤ 14 years of age according to Taipei City district, 1997–2007 (mean ± SD).

District	Pre-ADS weekdays	ADS event days	Post-ADS weekdays
Songshan		987.7 ± 280.3		995.3 ± 252.2		1008.7 ± 262.5
Daan		1467.7 ± 589.3		1484.1 ± 531.5		1504.9 ± 536.4
Datong		449.0 ± 185.6		419.9 ± 156.2		453.4 ± 177.0
Jhongshan		1326.8 ± 522.8		1343.1 ± 493.7		1356.7 ± 482.3
Neihu		1328.3 ± 513.1		1352.6 ± 423.3		1380.6 ± 454.2
Nangang		554.1 ± 202.6		572.8 ± 180.9		573.2 ± 186.2
Shihlin		1538.5 ± 562.3		1550.5 ± 492.4		1560.7 ± 512.4
Beitou		1305.6 ± 472.3		1299.0 ± 426.5		1318.1 ± 436.1
Sinyi		1038.2 ± 373.5		1033.4 ± 314.3		1056.8 ± 310.0
Jhongjheng		918.1 ± 375.0		945.4 ± 348.1		930.1 ± 359.6
Wanhua		915.5 ± 355.3		919.6 ± 308.4		938.8 ± 325.9
Wunshan		1284.6 ± 436.1		1272.9 ± 360.4		1310.1 ± 405.4
Clinic visits for respiratory disease were for all-cause respiratory diseases, including four major categories: upper respiratory infection, lower respiratory infection, asthma, and chronic obstructive pulmonary disease. Data are from the NHRI in Taiwan.						

Table 3 shows a consistently significant association between ADS and children’s respiratory clinic visits, especially in three ADS-related periods. The percentage of estimated rates of daily clinic visits for respiratory diseases increased by 2.54% (95% CI: 2.43, 2.66) during post-ADS weekdays compared with pre-ADS weekdays in preschool children, and by 5.03% (95% CI: 4.87, 5.20) in schoolchildren. In contrast, the percentage of estimated rates of clinic visits was significantly lower during ADS event days than for pre-ADS weekdays by –1.62% (95% CI: –1.71, –1.52) for preschool children and –5.66% (95% CI: –5.80, –5.53) for schoolchildren. Compared with pre-ADS weekdays, there were fewer visits during other days in preschool children (–0.25%; 95% CI: –0.32, –0.18) and more visits in schoolchildren (1.78%; 95% CI: 1.68, 1.87). For all children combined, estimated daily clinic visits increased on post-ADS weekdays and other days and decreased on ADS event days compared with pre-ADS weekdays. In general, regardless of the age stratification, daily rates of children’s clinic visits were highest on post-ADS weekdays and lowest during ADS event days.

**Table 3:** Percentage change in rates of daily clinic visits for respiratory conditions and spatial variance components in preschool children, schoolchildren, and all children combined in Taipei City, 1997–2007 [% (95% CI)].

Preschool children	Schoolchildren	All children
ADS episode						
Pre-ADS weekdays		Reference		Reference		Reference
ADS event days		–1.62 (–1.71, –1.52)		–5.66 (–5.80, –5.53)		–2.97 (–3.05, –2.90)
Post-ADS weekdays		2.54 (2.43, 2.66)		5.03 (4.87, 5.20)		3.38 (3.28, 3.47)
The other days		–0.25 (–0.32, –0.18)		1.78 (1.68, 1.87)		0.45 (0.39, 0.51)
Variance components						
σu2		0.0019		0.0025		0.0019
σs2		0.1576		0.0482		0.1184
ρ (%)		98.81		95.07		98.42
Notation: σu2 = unstructured spatial variance; σs2 = structured spatial variance; ρ = proportion of the structured spatial variance in total spatial variance, i.e., σs2/(σu2+ σs2) × 100%.						

**Figure 3:**
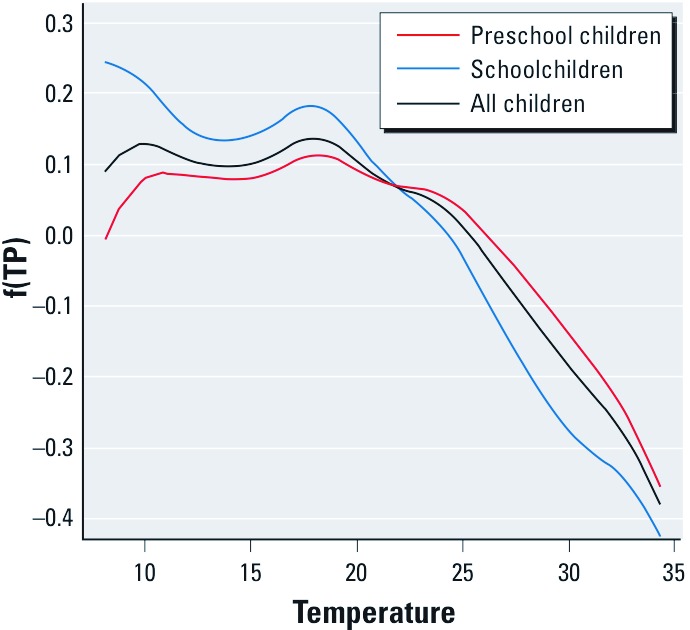
Temperature smoother for preschool children, schoolchildren, and all children. f(TP), amount of changes in children’s clinic visits explained by temperature variation.

We estimated that each 10-µg/m^3^ increase in PM_10_ was associated with a 1.18% (95% CI: 1.17, 1.19) increase in daily clinic visits for all children combined, and with estimated increases of 1.54% (95% CI: 1.52, 1.56) and 0.99%, 95% CI: 0.98, 1.01) in schoolchildren and preschool children, respectively. The association between temperature and children’s clinic visits for respiratory conditions was nonlinear but positive for colder temperatures and inverse for higher temperatures (Figure 3).

**Figure 4:**
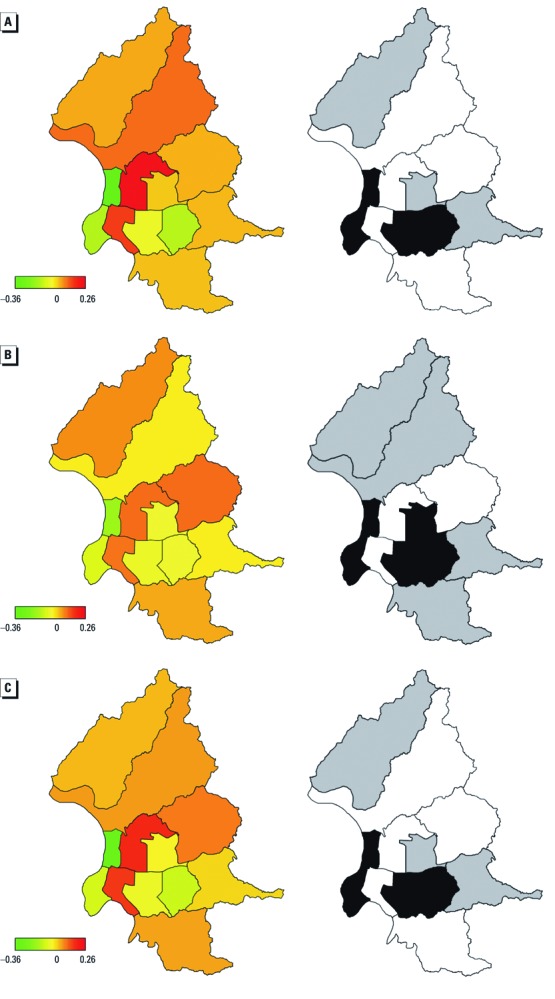
Structured spatial effect (left) with 80% posterior probability (right) for (*A*) preschool children, (*B*) schoolchildren, and (*C*) all children. Districts in black show strictly negative CIs, whereas white districts depict strictly positive CIs, and grey districts represent CIs containing zero.

We observed spatial heterogeneity in the RR of children’s respiratory clinic visits in Taipei City after adjusting for ADS events, PM_10_, and daily temperature (Figure 4). For preschool children, the highest RR appeared in the Jhongshan District at the center of Taipei City, three adjacent districts (Shihlin, Neihu, and Jhongjheng), and the Wunshan District (Figure 4A). For schoolchildren, the highest RR appeared in the Jhongshan, Jhongjheng, and Neihu Districts (Figure 4B). Relatively lower RR was detected in the Datong, Wanhua, and Shinyi Districts for preschool and schoolchildren. Structured spatial effects explained more of the geographic variation than unstructured spatial effects in both age groups, as indicated by the high ratios of variance components (ρ) > 95% (Table 3).

## Discussion

To our knowledge, this is the first population-based study examining the impact of ADS on children’s ambulatory clinic utilization for respiratory diseases. We found that the estimated rate of clinic visits in children ≤ 14 years of age was significantly increased during post-ADS weekdays compared with visits during pre-ADS weekdays. This finding is consistent with several studies in Taiwan. For example, health impacts of ADS have been estimated based on hospital admissions (Yang 2006; Yang et al. 2005a, 2005b, 2007, 2009), mortality (Bell et al. 2008; Chen et al. 2004), and emergency department visits (Chan et al. 2008). Inferences based on associations with more severe outcomes may be limited to vulnerable populations with deficits in respiratory function (Middleton et al. 2008). In contrast, the availability of the NHI database in Taiwan allowed us to estimate associations with clinic visits for respiratory conditions, which may provide a more comprehensive view of the health impact of ADS events in general populations. The results from the present study can inform governmental agencies trying to understand the health impact of ADS on the general population of children.

We found that schoolchildren appeared to be more adversely affected by ADS events than preschool children. One possible explanation could be differences in the extent of exposure. In Taiwan, schoolchildren are obligated to attend schools on weekdays even despite the occurrence of ADS. Although the idea of school suspension during severe ADS events has been proposed, no corresponding policy has been made. In contrast, preschool children may have been kept at home or indoors during ADS, resulting in less outdoor exposure.

Open access without provider choice restriction and small co-payments are features of Taiwan’s NHI program that encourage utilization of ambulatory and emergency services in Taiwan, where the average resident visits a clinic 14.2 times per year. As a result, NHI clinic visit data are highly likely to identify substantial variation in visits to medical centers. Geographic variation in visits among districts may be attributable to the absence of medical centers, such as those in the Sinyi and Nangang Districts. Medical facilities in the Datong and Wanhua Districts, the two oldest communities in Taipei, are relatively underdeveloped, without major medical centers in either district.

Study limitations include the potential for uncontrolled confounding—for example, related to holidays, influenza outbreaks, and weather. Temperature is strongly associated with influenza epidemics (Liao et al. 2009) because of the influence of temperature on influenza virus transmission patterns (Lowen et al. 2007). We used a natural cubic spline of temperature to adjust for changes in weather conditions over time, as others have done in previous studies (Bell et al. 2007, 2008; Dominici et al. 2006; Peng et al. 2005), using a variety of temperature measures including the average of three previous days’ temperature (Bell et al. 2007; Dominici et al. 2006). Continuous linear predictors or categorized variables are also frequently used to represent the temperature influence in statistical analysis (Yang et al. 2005a, 2007); however, the association between temperature and clinic visits was not explicitly linear in our study, as shown in Figure 3.

Holidays may play an important role in clinic visits because most clinics are closed during holidays. We adjusted for day of the week to account for changes in clinic use on Saturdays and Sundays. Few ADS events occurred during major holidays [New Year’s Day (1 January), Chinese New Year (5–9 days beginning on the last day of the 12th lunar month), Peace Memorial Day (28 February), Tomb-Sweeping Day (5 April), and Labor Day (1 May)]. Hence, we believe the impact of national holidays would not have substantially influenced our results.

Clinic visits recorded in the NHI data set included both walk-in visits and appointments; however, we believe that including appointments would not have biased associations with acute effects of ADS. In addition, about 70% of the total respiratory visits were for upper respiratory infections that were treated primarily at local clinics and hospitals during the study period. In Taiwan, patients are commonly seen at the local ambulatory services on a “first-come, first-served” basis, with relatively few appointments. Nonetheless, appointments may be more likely in medical centers or regional hospitals, which generally account for about 3% of all clinic visits for the relatively minor conditions that were most common in our data set, such as upper respiratory infections.

A major strength of the study was our use of the STAR modeling approach, which identifies temporal patterns of space–time processes based on both linear and nonlinear explanatory variables (Hastie and Tibshirani 1990). Moreover, using a spatial function in this study was an innovation in ADS and adverse human health research. In the STAR model, spatial heterogeneity can be revealed by modeling a spatial function and local information on the neighborhoods of interest. Both point-based (coordinate) and polygon-based (boundary) geographic data can be used in the STAR model (Fahrmeir and Kneib 2011), but we used boundary data because detailed addresses of patients were not available. Furthermore, the Bayesian framework of STAR model makes it possible to account for parameter uncertainty in the analysis. Use of this novel approach not only identified temporal changes in clinic visits across the entire city in association with ADS events, but also identified spatial patterns of clinic visits among Taipei districts that may reflect access to medical resources and the city transit system.

## Conclusions

The increased frequency of ADS in recent decades has raised concerns about health impacts on the general population of Taiwan, especially children. Our results show that clinic visits for respiratory conditions increased in children ≤ 14 years of age following ADS events, particularly in schoolchildren. Spatial patterns also show that most districts with significantly increased children’s respiratory clinic visits had large medical centers and hospitals, which suggests that children’s clinic visits in association with ADS events may be influenced by access to medical resources, especially medical centers. These results are relevant to policy makers responsible for protecting children’s health during ADS events, including governmental agencies considering policies for class suspension during ADS. Further study is required to estimate effects related to the magnitude of ADS events and ambient pollutant concentrations associated with ADS on the health of children.

## References

[r1] Adebayo SB, Fahrmeir L (2005). Analysing child mortality in Nigeria with geoadditive discrete-time survival models.. Stat Med.

[r2] Bates DV (1995). The effects of air pollution on children.. Environ Health Perspect.

[r3] Belitz C, Brezger A, Kneib T, Lang S (2009). BayesX - Software for Bayesian inference in structured additive regression models. Version 2.01.. http://www.stat.uni-muenchen.de/~bayesx.

[r4] Bell ML, Kim JY, Dominici F (2007). Potential confounding of particulate matter on the short-term association between ozone and mortality in multisite time-series studies.. Environ Health Perspect.

[r5] Bell ML, Levy JK, Lin Z (2008). The effect of sandstorms and air pollution on cause-specific hospital admissions in Taipei, Taiwan.. Occup Environ Med.

[r6] Berhane K, Gauderman WJ, Stram DO, Thomas DC (2004). Statistical issues in studies of the long-term effects of air pollution: The Southern California Children’s Health Study.. Statist Sci.

[r7] BNHI (Bureau of National Health Insurance) (2000). Regulations Governing Examination of Medical Care Services for National Health Insurance Medical Care Institutions, article 16, schedule II: Sampling examination and tracing method of medical care service cases.. http://www.nhi.gov.tw/webdata/webdata.aspx?menu=&menu_id=&wd_id=&webdata_id=2436.

[r8] Brezger A, Kneib T, Lang S. (2005). BayesX: Analysing Bayesian structured additive regression models.. J Stat Softw.

[r9] Centers for Disease Control and Prevention (2006). International Classification of Diseases, Ninth Revision, Clinical Modification (ICD-9-CM).. http://www.cdc.gov/nchs/icd/icd9cm.htm.

[r10] Chan CC, Chuang KJ, Chen WJ, Chang WT, Lee CT, Peng CM (2008). Increasing cardiopulmonary emergency visits by long-range transported Asian dust storms in Taiwan.. Environ Res.

[r11] Chen J-P (2002). Meteorological analysis and establishment of warning system of Asian dust storms. EPA-91-U1L1-02-108.

[r12] Chen YS, Sheen PC, Chen ER, Liu YK, Wu TN, Yang CY (2004). Effects of Asian dust storm events on daily mortality in Taipei, Taiwan.. Environ Res.

[r13] Cheng MT, Lin YC, Chio CP, Wang CF, Kuo CY (2005). Characteristics of aerosols collected in central Taiwan during an Asian dust event in spring 2000.. Chemosphere.

[r14] Choi JC, Lee M, Chun Y, Kim J, Oh S (2001). Chemical composition and source signature of spring aerosol in Seoul, Korea.. J Geophys Res.

[r15] Dockery DW, Pope CA, Xu X, Spengler JD, Ware JH, Fay ME (1993). An association between air pollution and mortality in six U.S. cities.. N Engl J Med.

[r16] Dominici F, Peng RD, Bell ML, Pham L, McDermott A, Zeger SL (2006). Fine particulate air pollution and hospital admission for cardiovascular and respiratory diseases.. JAMA.

[r17] Fahrmeir L, Kneib T (2011). Bayesian Smoothing and Regression for Longitudinal, Spatial and Event History Data.. New York:Oxford University Press.

[r18] Fahrmeir L, Lang S. (2001). Bayesian inference for generalized additive mixed models based on Markov random field priors.. J R Stat Soc Ser C (Applied Statistics).

[r19] Fromont A, Binquet C, Sauleau EA, Fournel I, Bellisario A, Adnet J (2010). Geographic variations of multiple sclerosis in France.. Brain.

[r20] Hastie T, Tibshirani R (1990). Generalized Additive Models.

[r21] Hong YC, Pan XC, Kim SY, Park K, Park EJ, Jin X (2010). Asian dust storm and pulmonary function of school children in Seoul.. Sci Total Environ.

[r22] Kandala NB, Brodish P, Buckner B, Foster S, Madise N (2011). Millennium development goal 6 and HIV infection in Zambia: what can we learn from successive household surveys?. AIDS.

[r23] Kazembe LN (2009). A Semiparametric Sequential Ordinal Model with Applications to Analyse First Birth Intervals.. Austrian J Statist.

[r24] Kazembe LN, Chirwa TF, Simbeye JS, Namangale JJ (2008). Applications of Bayesian approach in modelling risk of malaria-related hospital mortality.. BMC Med Res Methodol.

[r25] Kazembe LN Mpeketula PM 2010 Quantifying spatial disparities in neonatal mortality using a structured additive regression model. PLoS One 5 6 e1118 doi:10.1371/journal.pone.0011180 [Online 17 June 2010] PMC2887370 20567519

[r26] Kindermann R, Snell JL (1980). Markov Random Fields and Their Applications.

[r27] Lang S, Brezger A. (2004). Bayesian P-splines.. J Comput Graph Statist.

[r28] Liao CM, Chang SY, Chen SC, Chio CP (2009). Influenza-associated morbidity in subtropical Taiwan.. Int J Infect Dis.

[r29] Liu C-M, Young C-Y, Lee Y-C (2006). Influence of Asian dust storms on air quality in Taiwan.. Sci Total Environ.

[r30] Lowen AC, Mubareka S, Steel J, Palese P (2007). Influenza virus transmission is dependent on relative humidity and temperature.. PLoS Pathog.

[r31] Lu JF, Hsiao WC (2003). Does universal health insurance make health care unaffordable? Lessons from Taiwan.. Health Aff (Millwood).

[r32] Ma C-J, Kasahara M, Höller R, Kamiya T (2001). Characteristics of single particles sampled in Japan during the Asian dust-storm period.. Atmos Environ.

[r33] Middleton N Yiallouros P Kleanthous S Kolokotroni O Schwartz J Dockery DW 2008 A 10-year time-series analysis of respiratory and cardiovascular morbidity in Nicosia, Cyprus: the effect of short-term changes in air pollution and dust storms. Environ Health 7 39 doi:10.1186/1476-069X-7-39 [Online 22 July 2008] 18647382 PMC2517071

[r34] Musio M, Sauleau EA, Buemi A (2010). Bayesian semi-parametric ZIP models with space-time interactions: an application to cancer registry data.. Mathemat Med Biol.

[r35] National Aeronautics and Space Administration (2012). Rapid Response.. http://earthdata.nasa.gov/data/near-real-time-data/rapid-response.

[r36] Nee JB Chiang C-W Hu H-l Hu S-X Yu J-Y 2007 Lidar measurements of Asian dust storms and dust cloud interactions. J Geophys Res 112 D15 D15202 doi:10.1029/2007JD008476 [Online 3 August 2007]

[r37] Peng RD, Dominici F, Pastor-Barriuso R, Zeger SL, Samet JM (2005). Seasonal analyses of air pollution and mortality in 100 US cities.. Am J Epidemiol.

[r38] Pope CA, Burnett RT, Thun MJ, Calle EE, Krewski D, Ito K (2002). Lung cancer, cardiopulmonary mortality, and long-term exposure to fine particulate air pollution.. JAMA.

[r39] Salvi S. (2007). Health effects of ambient air pollution in children.. Paediatr Respir Rev.

[r40] Schwartz J. (2004). Air pollution and children’s health.. Pediatrics.

[r41] Taiwan Ministry of Interior (2000). Household Statistics.. Taipei, Taiwan.

[r42] Tseng CH (2004). Mortality and causes of death in a national sample of diabetic patients in Taiwan.. Diabetes Care.

[r43] TWEPA (Taiwan Environmental Protection Agency) (2011). Asian Dust Storm Monitoring Network.. http://dust.epa.gov.tw/dust/zh-tw/.

[r44] Yang CY (2006). Effects of Asian dust storm events on daily clinical visits for conjunctivitis in Taipei, Taiwan.. J Toxicol Environ Health A.

[r45] Yang C-Y, Chen C-C, Chen C-Y, Kuo H-W (2007). Air pollution and hospital admissions for asthma in a subtropical city: Taipei, Taiwan.. J Toxicol Environ Health, Part A.

[r46] Yang CY, Chen YS, Chiu HF, Goggins WB (2005a). Effects of Asian dust storm events on daily stroke admissions in Taipei, Taiwan.. Environ Res.

[r47] Yang CY, Cheng MH, Chen CC (2009). Effects of Asian dust storm events on hospital admissions for congestive heart failure in Taipei, Taiwan.. J Toxicol Environ Health A.

[r48] Yang CY, Tsai SS, Chang CC, Ho SC (2005b). Effects of Asian dust storm events on daily admissions for asthma in Taipei, Taiwan.. Inhal Toxicol.

[r49] Yu J-Y, Liu K-Y (2003). Update and Application of Asian Dust Storm Database. EPA–92–L105–02–207.

[r50] Zhou M, Okada K, Qian F, Wu PM, Su L, Casareto BE (1996). Characteristics of dust-storm particles and their long-range transport from China to Japan—case studies in April 1993.. Atmos Res.

